# The neutrophil protein S100A12 is associated with a comprehensive ultrasonographic synovitis score in a longitudinal study of patients with rheumatoid arthritis treated with adalimumab

**DOI:** 10.1186/1471-2474-15-335

**Published:** 2014-10-04

**Authors:** Hilde Haugedal Nordal, Johan G Brun, Anne-Kristine Halse, Roland Jonsson, Magne K Fagerhol, Hilde Berner Hammer

**Affiliations:** Broegelmann Research Laboratory, Department of Clinical Science, University of Bergen, Postboks 7804, N-5020 Bergen, Norway; Section for Rheumatology, Department of Clinical Science, University of Bergen, Bergen, Norway; Department of Rheumatology, Haukeland University Hospital, Bergen, Norway; Prof. Fagerhol’s Research Laboratory, Oslo, Norway; Department of Rheumatology, Diakonhjemmet Hospital, Oslo, Norway

**Keywords:** S100 proteins, S100A12, Ultrasonography, Rheumatoid arthritis, Inflammation, Biologic therapy

## Abstract

**Background:**

The calcium-binding protein S100A12 correlates with measures of disease activity in patients with rheumatoid arthritis (RA). The protein reflects neutrophil activation and the present objective was to explore in a pilot study the associations between S100A12 and other inflammatory markers, clinical assessments as well as degree of synovitis detected by a comprehensive ultrasonography (US) examination in RA patients during biologic treatment.

**Methods:**

Twenty patients with RA were examined clinically and by use of US as well as laboratory markers S100A12, calprotectin, C-reactive protein (CRP) and erythrocyte sedimentation rate (ESR) before starting adalimumab, with follow-up after 1, 3, 6 and 12 months. Ultrasonographic B-mode (BM) and power Doppler (PD) assessments of 78 joints, 36 tendons/tendon groups and 2 bursas were performed, and sum US scores calculated. Wilcoxon signed rank test assessed treatment response and Spearman rank correlation test was used to calculate correlations.

**Results:**

The concentrations of S100A12 decreased after 3 months (p < 0.01) and significant correlations were found between S100A12 and the other laboratory markers during follow-up (0.50-0.62, p < 0.05). Of the clinical assessments, S100A12 had highest correlations with the assessor’s global VAS (0.46-0.85, p < 0.05). Compared with CRP and ESR, S100A12 showed higher correlations with the sum US scores (both BM and PD), with median (range) correlation coefficients of 0.55 (0.35-0.78 (NS-p < 0.001)) for sum BM scores and 0.45 (0.27-0.75 (NS-p < 0.001)) for sum PD scores.

**Conclusions:**

The S100A12 protein was significantly associated with other inflammatory markers, clinical assessments as well as sum US scores, indicating that S100A12 is a potential marker of inflammation in RA patients.

**Electronic supplementary material:**

The online version of this article (doi:10.1186/1471-2474-15-335) contains supplementary material, which is available to authorized users.

## Background

Rheumatoid arthritis (RA) is characterized by synovitis that untreated may lead to joint damage and functional disability. Even in clinical remission, a majority of patients with RA had ongoing synovial inflammation as detected by magnetic resonance imaging (MRI) or ultrasonography (US) [1]. Use of these sensitive imaging modalities is expensive or time consuming, thus sensitive biomarkers reflecting the ongoing joint inflammation may be of considerable value for clinical decisions.

S100A12 is a calcium-binding protein, expressed predominantly in neutrophils, but also in monocytes [2, 3]. It is released from activated neutrophils [4] and has proinflammatory effects on endothelium and immune cells that have been summarized in a review article [5].The protein was up-regulated in synovial fluid in RA patients compared with patients with osteoarthritis [6], and it differentiated RA from other forms of inflammatory arthritis when exploring synovial fluid [7]. S100A12 was increased in serum of RA patients compared with healthy controls, and was associated with the presence of rheumatoid factor (RF) and anti-citrullinated peptide antibodies (ACPA) [8]. Both in synovial fluid and in serum elevated levels of S100A12 were found in erosive forms of RA compared with non-erosive forms [9]. S100A12 was found in inflamed synovia of RA patients, but it was not expressed in synovial tissue without inflammation [10]. Serum concentrations of S100A12 correlated with synovial fluid levels and with measures of disease activity as erythrocyte sedimentation rate (ESR) and Ritchie articular index [10]. S100A12 serum concentrations were also elevated in RA patients before treatment with intra-articular corticosteroids or anti-tumour necrosis factor (anti-TNF), and decreased significantly in patients who responded to these treatments [11]. S100A12 expression in synovia was also reduced in these two groups after treatment [11]. Although serum levels of S100A12 are increased in RA patients compared with controls [8, 10], the upper normal level in healthy persons has yet to be defined.

US is a sensitive imaging modality for detecting synovitis in RA patients. The amount of synovitis is detected by B-mode (BM). The degree of vascularization, reflecting the inflammatory activity, is evaluated by use of power Doppler (PD). A comprehensive US examination of joints and tendons of RA patients may thus be able to reflect the total amount of ongoing inflammation in these structures.

The major leukocyte S100 protein, calprotectin (hetero-complex of S100A8/A9), was investigated earlier in plasma samples of this cohort and correlated with the US sum scores [12]. The present pilot study explores the associations between serum levels of S100A12 and laboratory and clinical assessments of disease activity, as well as US sum scores from a comprehensive examination of joints and tendons in a longitudinal follow-up of RA patients starting biologic treatment.

## Methods

### Patients

Twenty patients (15 women and 5 men) with RA according to the American Rheumatism Association 1987 revised criteria [13] with median (range) age 53 (21 to 78) years and disease duration 7.5 (1 to 26) years were included as described elsewhere [14]. A total of 70% were IgM RF positive and 80% ACPA positive. At inclusion all patients used methotrexate as disease-modifying anti-rheumatic drug (DMARD) (median (range) dose 17.5 (7.5-25) mg per week), 70% used prednisolone (median (range) dose 7.5 (3.75-15) mg per day) and two patients were on daily non-steroidal anti-inflammatory drugs (NSAIDs). Median (range) DAS28 was 5.3 (3.4-7.7) at baseline. The patients were included the day they started treatment with adalimumab 40 mg every second week as their first biologic agent. The study was approved by the Regional Committee for Medical and Health Research Ethics, South-East (REK), and the patients gave written consent according to the Declaration of Helsinki.

### Laboratory analyses

Blood samples were drawn at inclusion and after 1, 3, 6 and 12 months of treatment with adalimumab. Conventional inflammatory markers included ESR and CRP, analysed by use of in-house methodology, with upper normal levels of 20 mm/h for ESR and 4 mg/L for CRP. Calprotectin in plasma was examined by use of enzyme-linked immunosorbent assay (ELISA) kits from CALPRO AS, Norway. Sera from all five examinations were frozen at −70°C. S100A12 concentrations in serum were determined by ELISA as follows: 96-well high binding Costar 3590 micro plates from Costar Corporation, USA, were coated with mouse monoclonal anti-S100A12 5 μg/mL in 0.1 M sodium citrate pH 6, 150 μL per well, for 18 h to 4 weeks at +5°C. Samples were diluted 1:5 using the sample dilution buffer. After washing the wells three times, 50 μL of calibrators or samples were added in triplicate wells and incubated at room temperature with shaking (500 rpm) for 40 min. A second monoclonal was conjugated with alkaline phosphatase. After washing again, all wells were added 50 μL of the ALP conjugate diluted 1:1000 and incubated again for 40 min. After a final wash, all wells were given 100 μL ALP substrate. Reading at 405 nm was performed when the optical density (OD) of the higher standard (512 ng/mL) was between 2.0 and 3.0. For samples with concentrations of 512 and 16 ng/mL the intra-assay coefficients of variation were 6.5% and 10.6% and inter-assay coefficients of variation were 2.3% and 10.9% respectively. The lower limit of quantification of the ELISA was 4 ng/mL. The reactivity and specificity of the antibodies were previously tested by ELISA using micro wells coated with S100A12 or calprotectin, which has considerable amino acid sequence homology to S100A12 [15].

### Clinical assessments

Forty joints (proximal inter-phalangeals 1–5, metacarpo-phalangeals 1–5, wrists, elbows, shoulders, knees, ankles and metatarso-phalangeals 1–5) were assessed by one of two experienced nurses for tenderness and swelling, blinded for the results of the US examination. They also scored the global disease activity on a visual analogue scale (assessor’s global VAS). The disease activity score DAS28 [16] was calculated.

### Ultrasonography

US examinations were performed by one experienced sonographer (HBH using a Siemens Antares Sonoline machine; Siemens Medical Solutions, California, USA) at the day of inclusion and after 1, 3, 6 and 12 months as previously described [12, 14]. A total of 78 joints and 36 tendons or tendon groups as well as the bilateral subdeltoid bursae were assessed for arthritis, tenosynovitis and bursitis by grey scale or B-mode (BM) presence of synovial hypertrophy and fluid (scored together) and presence of power Doppler (PD) vascularization, both scored on a 4–point scale (0 = no, 1 = minor, 2 = moderate, 3 = major presence). The US examiner had no access to US results from the previous examinations and was blinded for the results from the clinical joint assessments and laboratory tests completed the same day.

### Statistics

Wilcoxon signed rank test was used to analyse the differences between the levels of S100A12, ESR, CRP and US scores before starting adalimumab treatment and during follow-up. Associations between laboratory markers, clinical evaluations and US scores were analysed by use of Spearman rank order correlations. IBM SPSS Statistics version 20 was used for the statistical analyses and p < 0.05 was considered statistically significant. All tests for significance were two-sided.

## Results

**Figure 1:**
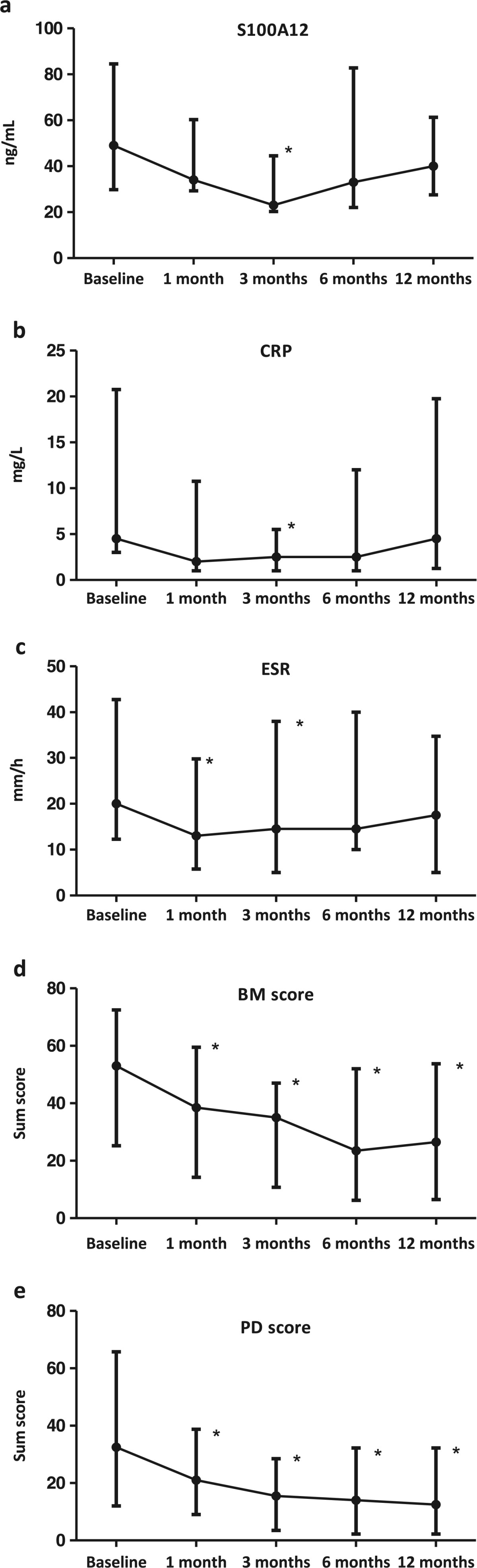
**Median levels (interquartile range) of the inflammatory markers and ultrasonographic scores during 12 months follow-up. a)** Concentrations of S100A12. **b)** Concentrations of C-reactive protein (CRP). **c)** Erythrocyte sedimentation rates (ESRs). **d)** B-mode (BM) sum scores. **e)** Power Doppler (PD) sum scores. *Significantly decreased levels from baseline, p<0.01.

The patients had median (range) S100A12 levels of 49 (18 to 475) ng/mL at baseline, 34 (18 to 687) ng/mL after 1 month, 23 (16 to 173) ng/mL after 3 months, 33 (19 to 394) ng/mL after 6 months and 40 (12 to 620) ng/mL after 12 months. The level of S100A12, ESR and CRP changed significantly from baseline to 3 months follow-up and ESR already after 1 month, while the sum US scores decreased significantly during follow-up (Figure 1).

There were significant correlations between S100A12 levels and sum US scores of all joints, tendons and bursae for BM and PD at baseline, after one month and 6 months. During the study, S100A12 showed higher correlation coefficients with the US scores than the conventional inflammatory parameters CRP and ESR (Table 1). The correlations between S100A12 and the inflammatory markers, as well as clinical assessments, are shown in Table 2. Closest associations were found between S100A12 and CRP/assessor’s global VAS. During follow-up, different degree of correlation was found between the leukocyte proteins S100A12 and calprotectin, from not significant to moderately significant correlations.

**Table 1:** **Spearman rank correlation coefficients between inflammatory markers and sum BM/PD scores**

	Baseline	1 month	3 months	6 months	12 months
S100A12	0.78***/0.75***	0.68***/0.65***	0.35/0.27	0.55*/0.45*	0.43/0.43
ESR	0.37/0.48*	0.36/0.35	0.45*/0.42	0.32/0.33	0.35/0.40
CRP	0.33/0.34	0.04/0.11	0.49*/0.35	0.59**/0.43	0.57**/0.55*

CRP = C-reactive protein, ESR = erythrocyte sedimentation rate, BM = B-mode, PD = power Doppler, *p < 0.05, **p < 0.01, ***p < 0.001.

**Table 2:** **Spearman rank correlation coefficients between S100A12 and inflammatory markers/clinical evaluations of disease activity**

	Baseline	1 month	3 months	6 months	12 months
ESR	0.50*	0.29	0.25	0.38	0.62**
CRP	0.61**	0.40	0.50*	0.66**	0.59**
Calprotectin	0.56*	0.34	0.12	0.52*	0.57**
Assessor’s global VAS	0.85***	0.58**	0.12	0.52*	0.46*
Swollen joints (of 40)	0.41	0.45*	0.33	0.31	0.48*
DAS28	0.39	0.21	0.23	0.34	0.51*

ESR = erythrocyte sedimentation rate, CRP = C-reactive protein, VAS = visual analogue scale, DAS28 = disease activity score of 28 joints, *p < 0.05, **p < 0.01, ***p < 0.001.

## Discussion

The present one-year follow-up study of RA patients starting treatment with adalimumab is the first to analyse the associations between serum levels of S100A12 and a comprehensive US joint examination, and significant correlations were currently found.

All the inflammatory laboratory markers decreased during the study. However, they tended to increase at 6 months onward (Figure 1), which could indicate reduced efficacy of the medication. It could be speculated that it may be caused by development of antibodies against adalimumab in some of the patients [17]. On the other hand, this tendency was not seen for the US sum scores [14]. A possible explanation could be that rise in laboratory markers may be seen before changes in US scores. It could also be speculated that, in parallel with findings which suggest a possible dissociation between disease activity and radiographic damage [18], there could be a dissociation between the disease activity as measured by inflammatory markers and US findings in some patients treated with a biologic agent.

The S100A12 serum levels were lower in this study than those reported for RA patients with active arthritis [10, 11]. The decrease following successful therapy was also less pronounced than what has been reported previously [11]. This might be explained by the use of a different ELISA-test and thus the levels cannot be directly compared. In addition, the levels of the other inflammatory markers were rather low, even at baseline.

S100A12 correlated better and more consistently with the comprehensive US sum BM and PD scores than the conventional inflammatory parameters (Table 1). However, this might be partly explained by the low levels of the conventional parameters, at least for CRP having a median level of 4.5 mg/L at baseline. US is a sensitive modality for detection of synovitis, and thus the present findings indicate that the neutrophil protein S100A12 is a biomarker of synovial inflammation in RA. Presently small and large joints were equally weighted in the US scores, and thus not exactly reflecting the total amount of synovitis. A larger study should include a weighting of joints taking into account the size of the inflamed synovium. This might be a more accurate way of assessing the associations between US and the leukocyte proteins calprotectin and S100A12.

Although both S100A12 and calprotectin are released by neutrophil activation, their correlations during follow-up were presently found to be variable. S100A12 was not superior to calprotectin as a biomarker for RA in this cohort, but the associations between these two S100 proteins and clinical assessments and US scores should be explored in further studies. Also S100A12’s ability to predict destruction should be assessed in larger cohorts of RA patients.

In a large cohort of patients with juvenile idiopathic arthritis it was found that increased levels of S100A12 and calprotectin, in addition to high-sensitivity C-reactive protein, indicated subclinical inflammation and thereby could identify patients with increased risk of relapse when in clinical remission [19]. Whether this is paralleled in RA, remains to be shown. Many RA patients in clinical remission have remaining inflammatory activity of their joints detected by US [1]. In our cohort a significantly higher number of inflamed joints were found by US than by clinical assessments at all the examinations [14]. Serum levels of S100A12 may be of help to identify RA patients with remaining inflammatory activity of joints.

The present study has several strengths. Primarily, this is the first study exploring the associations between S100A12 serum levels and a comprehensive US examination. In addition, it is a longitudinal 12 months follow-up study, giving the possibility to study changes in S100A12 during treatment with biologic medication. The weakness of this pilot study is the limited number of patients included, which reduces the strength of the present findings.

## Conclusions

In this pilot study of 20 RA patients followed for 12 months after initiation of adalimumab treatment, serum levels of S100A12 were associated with clinical and laboratory assessments as well as with a comprehensive ultrasonographic examination. This indicates that S100A12 could be a biomarker of synovitis in RA, and these promising findings should be explored further in larger cohorts of RA patients to evaluate S100A12 as a marker of inflammation.

## Authors’ original submitted files for images

Below are the links to the authors’ original submitted files for images. Authors’ original file for figure 1

## Abbreviations

ACPA: Anti-citrullinated antibodies
BM: B-mode or grey scale
CRP: C-reactive protein
DAS28: Disease activity score of 28 joints
ELISA: Enzyme-linked immunosorbent assay
ESR: Erythrocyte sedimentation rate
MRI: Magnetic resonance imaging
PD: Power Doppler
RA: Rheumatoid arthritis
TNF: Tumour necrosis factor
US: Ultrasonography
VAS: Visual analogue scale.
