# Supplementary figures and images for: The neutrophil protein S100A12 is associated with a comprehensive ultrasonographic synovitis score in a longitudinal study of patients with rheumatoid arthritis treated with adalimumab
Source: BMC Musculoskelet Disord. 2014 Oct 4;15:335. doi: 10.1186/1471-2474-15-335 (PMC4196044; doi:10.1186/1471-2474-15-335)

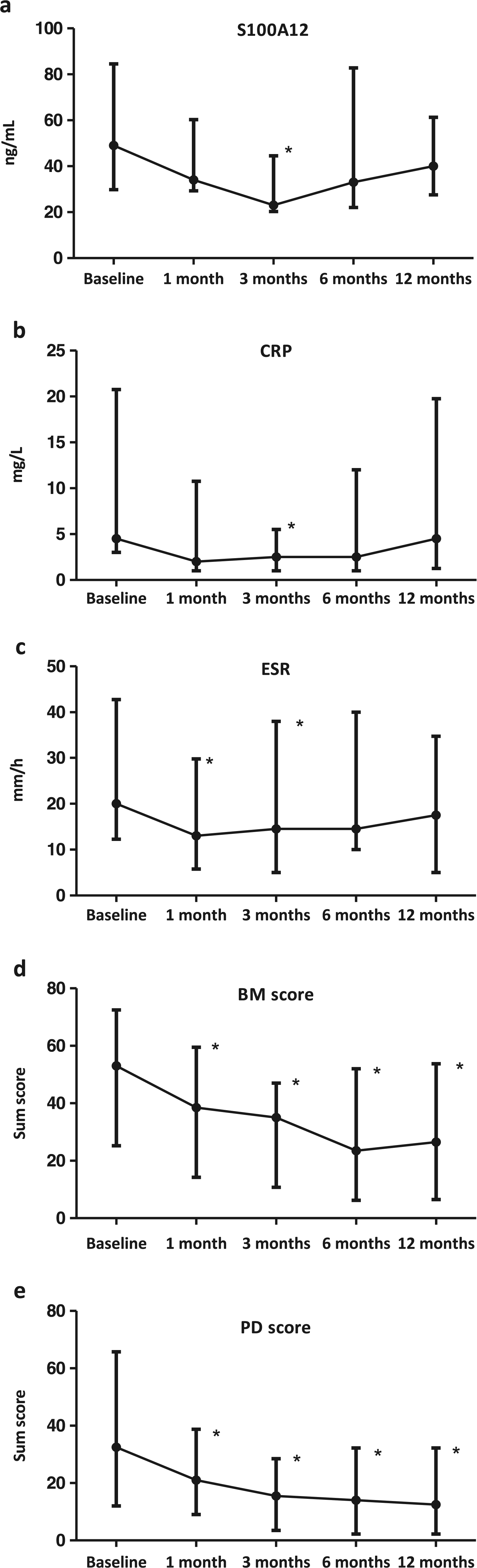

Supplement: Supplementary file 1 — Authors’ original file for figure 1 [file 12891_2014_2271_MOESM1_ESM.tif]
